# Novel sternoclavicular hook plate for the treatment of posterior sternoclavicular dislocation: a retrospective study

**DOI:** 10.1186/s13018-023-04436-7

**Published:** 2023-12-09

**Authors:** Hanlong Xin, Xingui Wang, Shaohua Zhang, Lie Lin, Haixiao Chen, Huaxing Hong

**Affiliations:** 1 Department of Orthopaedic Surgery, Taizhou Hospital of Zhejiang Province Affiliated to Wenzhou Medical University, Taizhou, China; 2 Intensive Care Unit (ICU), Taizhou Hospital of Zhejiang Province Affiliated to Wenzhou Medical University, Taizhou, China

**Keywords:** Sternoclavicular hook plate, Posterior sternoclavicular dislocation, Internal fixation, Surgical treatment, Joint functional recovery

## Abstract

**Background:**

Controversies regarding the optimal internal fixation method for posterior sternoclavicular dislocation (SCD) exist. Therefore, this study aimed to investigate the clinical efficacy of a new type of sternoclavicular hook plate for treating posterior SCD.

**Methods:**

Eleven patients (eight men and three women) with posterior SCD who underwent treatment with the new sternoclavicular hook plate from June 2011 to January 2022 were retrospectively analyzed. The patients’ ages ranged from 33 to 71 years (54.91 ± 13.58 years). Operation time, blood loss, length of hospital stay, and postoperative complications were recorded. Postoperative joint reduction and healing were evaluated using radiography and computed tomography. The Constant–Murley and Rockwood sternoclavicular joint scores were used to evaluate the functional recovery of the affected limb 12 months after surgery.

**Results:**

All 11 patients were followed up for 12–24 months (18.00 ± 3.74 months). All incisions healed by first intention. The healing time ranged from 9 to 13 days (10.82 ± 1.54 days), and the joint healing time was 3–4 months (3.55 ± 0.52 months). The operation time was 45–75 min (59.55 ± 11.06 min), intraoperative blood loss was 22–58 mL (39.91 ± 11.07 mL), and the length of hospitalization was 6–14 days (9.91 ± 3.27 days). There were no complications such as infections, internal fixation failure, or nerve injury. The Constant–Murley score was 93.64 ± 9.01 at 12 months postoperatively. The Rockwood score was 13.36 ± 1.86, of which nine cases were excellent, one case was good, and one case was fair.

**Conclusion:**

The novel sternoclavicular hook plate is effective for the treatment of posterior SCD. This novel device can facilitate early joint functional exercises and good functional recovery.

## Introduction

Sternoclavicular dislocation (SCD) accounts for approximately 1% of thoracic shoulder joint dislocations [1], and posterior SCD is a relatively rare dislocation compared to anterior dislocation [2]. SCD is often caused by high-energy trauma that usually compresses and damages important tissues, such as nerve vessels and the trachea, near the back of the joint. In addition, acute posterior dislocation should be treated as an emergency as 30% of patients experience mediastinal injury [3]. Congestion of the neck and ipsilateral upper limbs, difficulty swallowing, coughing, hoarseness, or suffocation, which suggest probable mediastinal obstruction, are potential life-threatening emergencies. This type of dislocation often requires high-risk surgical treatment.

Commonly used surgical treatment methods for SCD include Kirschner wire or wire fixation, plate fixation, medial clavicle resection, and sternoclavicular arthrodesis [4]. Kirschner wire or wire fixation may cause complications, such as damage to blood vessels and important posterior organs, which are caused by the fracture or displacement of implants [5]. Using a locking plate or a T-shape plate fixed to the manubrium and the clavicle with screws can result in a loss of amphiarthrodial function of the sternoclavicular joint [6]. Therefore, controversies regarding the optimal internal fixation method for posterior SCD remain [7].

The sternoclavicular hook plate (patent no. ZL2003201079412) is an internal fixation material designed and developed by our department for the treatment of traumatic SCD that results in safe and reliable fixation and can also retain the amphiarthrodial function of the sternoclavicular joint [8]. Whether the treatment of posterior SCD with the sternoclavicular hook plate can achieve satisfactory results remains unknown. This study retrospectively analyzed the clinical data of 11 patients with posterior SCD treated with the novel sternoclavicular hook plate for internal fixation between June 2011 and May 2022, with the aim of exploring the clinical efficacy of the sternoclavicular hook plate in treating posterior SCD.

## Materials and methods

### Patients

This study included eight men and three women aged 33–71 years (54.91 ± 13.58 years) (Table 1). The inclusion criteria were as follows: (1) age > 18 years; (2) patients with posterior SCD as indicated by radiography and computed tomography (CT); (3) closed injury without vascular or nerve injury; (4) internal fixation performed using a thoracic locking hook plate; and (5) complete follow-up time > 1 year, disease calendar, and imaging data. The exclusion criteria were as follows: (1) patients with severe underlying medical diseases who could not tolerate anesthesia or surgery; (2) previous shoulder disease and trauma history; and (3) pathological fracture.

**Table 1:** Patient characteristics (n = 11)

	Value
Age, means (range), y	54.91 (33–71)
Sex, male/female, n	8/3
Side, left/right SCD	2/9
Time of follow-up, means(range), m	18 (15–24)
Mechanism of injury, n	
Motor vehicle accident	4
Hit by heavy objects	2
Fall by biking	2
Fall from height	1
Fall to the ground	1
Brawl	1

### Surgical materials

**Fig. 1:**
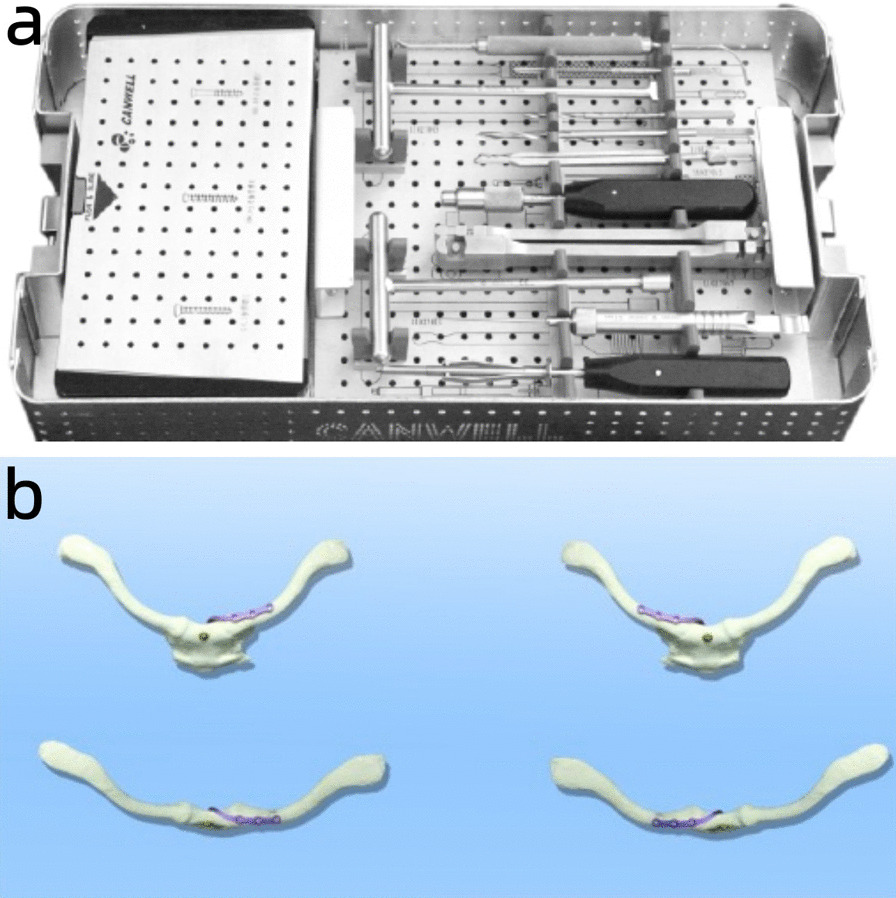
**a** 21 special tools to meet surgical needs; **b** Placement of the sternoclavicular joint hook

**Fig. 2:**
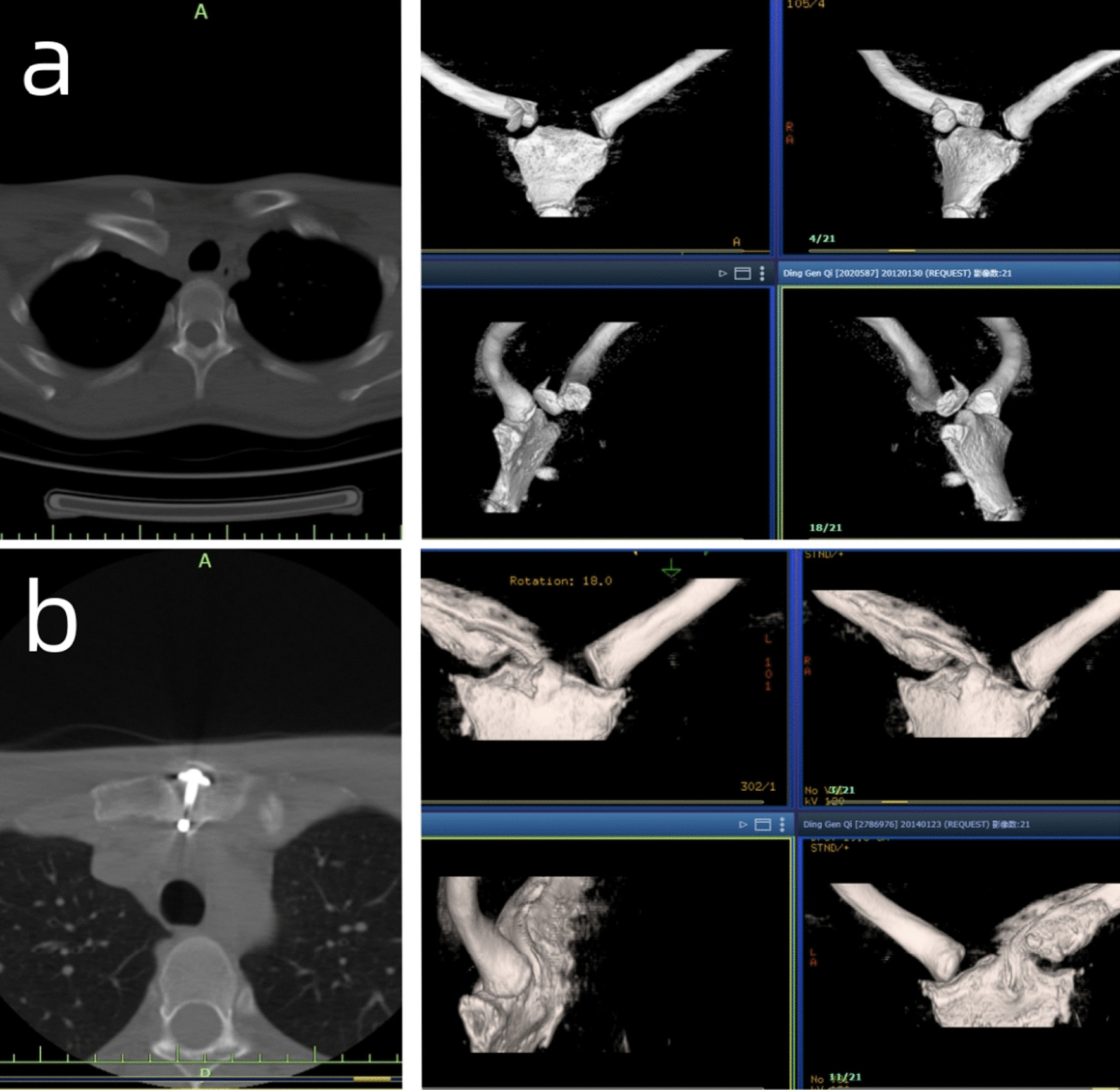
**a** CT image of a 33-year-old male patient with posterior dislocation of left sternoclavicular joint preoperatively; **b** The plate and joint was restored to normal 2 years postoperatively

Based on the anatomical characteristics of the human sternoclavicular joint, an anatomical sternoclavicular hook plate (21 mm/24 mm, two kinds of height, 3/4 hole) and 21 special supporting instruments (patent numbers ZL2003201079412 and L201220667654.6) were designed and completed (Fig. 1). Compared to the traditional plate, the new plate has a double thread nut lock and pre-bent overhang beam. Furthermore, a designed hook was inserted from the posterior upper edge of the manubrium and was hooked by drilling into the manubrium to act as a lever pivot. Pushing down and pulling can reduce anterior and posterior dislocation, respectively. For anterior dislocation, only a standard sternoclasp plate was used. For posterior dislocation, nuts and spacers were added to the head of the plate hook and at the front of the manubrium to prevent postoperative re-dislocation (Fig. 2).

### Surgical method

**Fig. 3:**
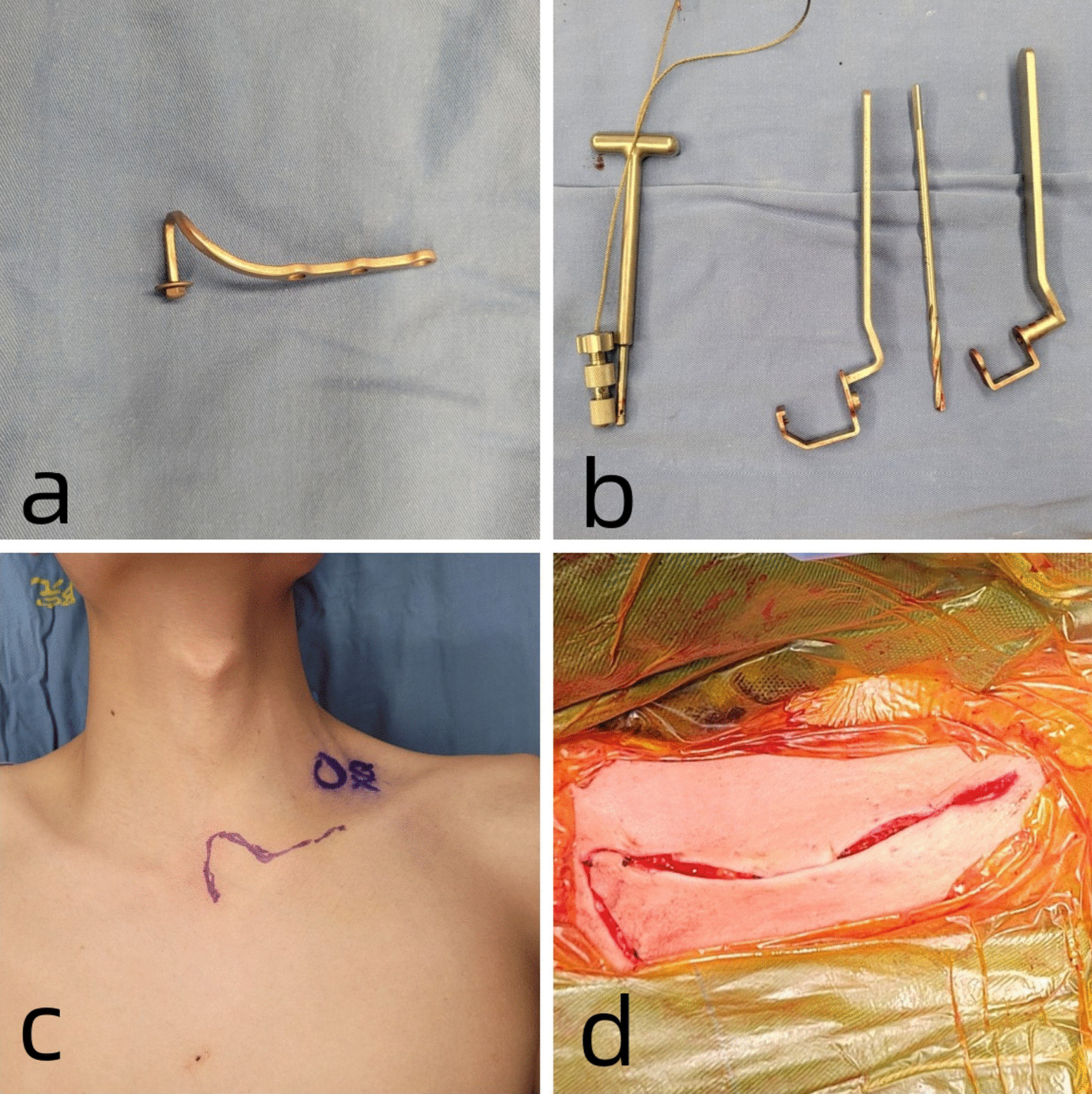
**a**, **b** Sternoclavicular hook plate and its accompanying tools; **c**, **d** Posterior dislocation of the lift sternoclavicular joint with a 7-shaped incision

**Fig. 4:**
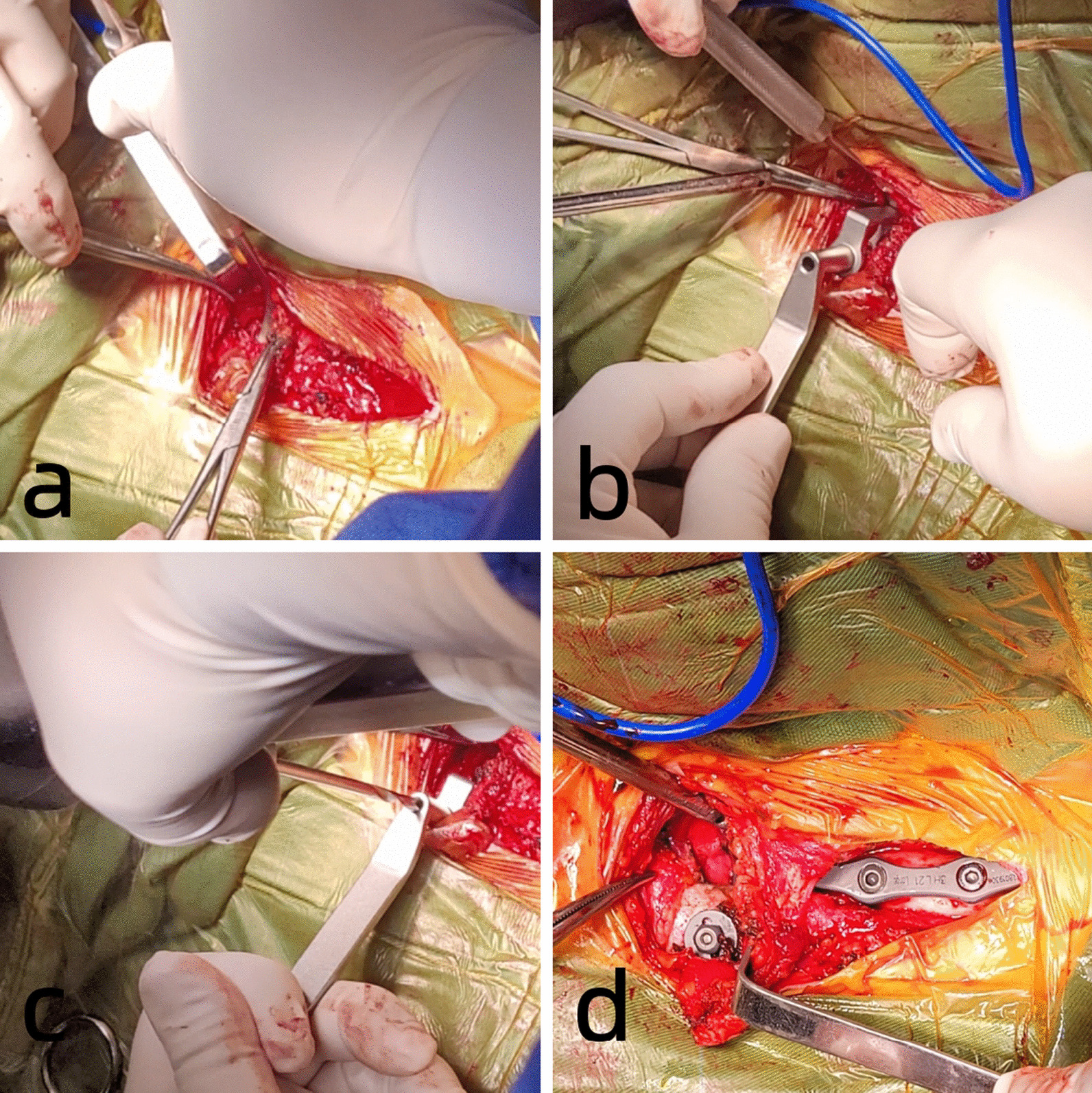
**a** Soft tissue of the upper edge of the sternal manubrium is stripped by a special periosteal elevator; **b** A special “C” shaped protective sleeve is inserted from above the sternal manubrium; **c** There is a barrier sheet at the posterior sleeve, which protect the important tissues under the sternal manubrium during the drilling; **d** Gaskets and nuts should be used during a posterior dislocation of the sternoclavicular joint to prevent postoperative redislocation

Endotracheal intubation was performed under general anesthesia. The patient was placed in the supine position with the interscapular area slightly elevated to slightly abduct the bilateral shoulders and extend the neck [9]. A 7-shaped incision measuring 8–10 cm centered on the injured side of the sternoclavicular joint was made from the proximal clavicle to the upper margin of the manubrium, exposing the proximal clavicle and the upper part of the manubrium (Fig. 3). The lateral sternal insertion of the sternocleidomastoid muscle was protected. A special periosteal elevator separated the appropriate space from top to bottom along the posterior part of the manubrium sternum, and a “C” shaped protective sleeve was then inserted. The sternal orifice was prepared by drilling from front to back, and a 3-hole thoracic hook plate was selected to wind out from the posterior side of the manubrium toward the anterior orifice. The steel plate reduced the chances of fracture and dislocation by lifting, and a gasket and nut were added to the thread of the hook head to prevent posterior dislocation. Intraoperative C-arm X-ray fluoroscopy showed that the proximal clavicle fracture and SCD were well reduced, and the internal fixation plate was in a good position. The incision was rinsed, hemostasis was achieved, and the incision was sutured layer-by-layer (Fig. 4).

### Efficacy evaluation criteria

The efficacy evaluation criteria were adopted using the Constant–Murley grading [10] and Rockwood et al. [11] chest lock section scores for functional evaluation. The Constant–Murley score includes pain, daily living ability, range of motion (ROM), and strength. The Rockwood score includes pain, ROM, muscle strength, daily routine activity limitations, and subjective results. The full score was 15; a total score < 7 was poor, 7–9 was fair, 10–12 was good, and 13–15 was excellent.

### Postoperative management

On the first postoperative day, cefuroxime sodium (1.5 g twice daily) was administered as prophylactic anti-infection therapy. On the second postoperative day, X-rays of the operated clavicle and CT images of the sternoclavicular joint were reviewed. After pain relief, shoulder pendulum, passive flexion, and upward lifting of the affected limb were performed. The dressing was changed once every 2–3 days, stitches were removed 2 weeks later, and active shoulder flexion, extension, and abduction exercises were started 3 weeks later. Severe pushing, pulling, and lifting were avoided for 3 months. Radiography and CT images were reviewed regularly.

## Results

All 11 patients were followed up for 12–24 months (18.00 ± 3.74 months). The operation time was 45–75 min (59.55 ± 11.06 min), intraoperative blood loss was 22–58 mL (39.91 ± 11.07 mL), and length of hospitalization was 6–14 days (9.91 ± 3.27 days). On the second postoperative day, the dislocation was re-examined using radiography and CT. All incisions healed by first intention. The healing time was 9–13 days (10.82 ± 1.54 days), and there were no complications such as vascular or nerve injury and pneumothorax. The joint healing time was 3–4 months (3.55 ± 0.52 months), and there were no complications such as infections, internal fixation failure, and nerve injury. All thoracic hook plates were removed 12–18 months postoperatively. The Constant–Murley score was 93.64 ± 9.01 at 12 months after the operation. The pain score was 14.09 ± 2.02, daily living ability score was 18.18 ± 2.56, ROM score was 36.36 ± 4.80 points, and strength was 25 points. The Rockwood score was 13.36 ± 1.86, pain was 2.82 ± 0.41, ROM was 2.27 ± 0.65, muscle strength was 3, restriction of daily activities was 2.55 ± 0.52, and the subjective result was 2.73 ± 0.65, of which nine cases were excellent, one case was good, and one case was fair.

## Discussion

### Characteristics of posterior SCD and internal fixation

The sternoclavicular joint is the only true joint between the upper shoulder strap and the trunk [12]. The clavicular notch of the manubrium is less than half the inner end of the clavicle; therefore, it is potentially uncoordinated and unstable. Its stability mainly depends on the reinforcement of the surrounding ligaments, which makes it the smallest bone-stable joint in the body. SCD is a relatively rare dislocation in clinical practice [13]. It is mostly caused by a large external force applied directly to the anterior part of the clavicle, pushing the proximal part of the clavicle behind the sternum and resulting in posterior SCD. Posterior dislocations are less common than anterior dislocations [2, 14]. However, because of the proximity of important structures to the medial posterior clavicle, accurate identification of posterior dislocations is important. Diagnosis is based on three aspects: medical history, clinical manifestations, and auxiliary examination.

Some studies [15, 16] have reported that early surgical treatment was adopted for posterior SCD, and that open reduction and internal fixation became the preferred treatment for this fracture type for young patients and patients requiring high activity [17]. While there are numerous surgical techniques, including large Kirschner wire, cannulated screws, suture anchor fixation or plate fixation [18–22], it is worth noting that operative methods utilizing metallic pin fixation with K-wires, Steinmann pins, and threaded pins with bent ends are associated with severe complications, including fatalities, across all age groups and are now contraindicated [23]. Georgios et al. used suture buttons to reduce and fix the sternoclavicular joint, with one button on the superior surface of the medial end of the clavicle and the other button on the anterior surface of the sternum; function was good at the 12-month follow-up. The use of suture buttons was also reported for superior dislocation, but its efficacy on posterior sternoclavicular joint dislocation remains uncertain [24]. Wire cerclage techniques were used to stabilize sternoclavicular joints during open reduction, resulting in satisfactory shoulder motion range. However, chronic mild pain was reported [25]. Alternative approaches involve resecting the medial part of the clavicle [21, 26] and employing various soft tissue methods for costoclavicular ligament reconstruction, such as suture repair of the costoclavicular or sternoclavicular ligaments [27], costoclavicular tenodesis using the subclavius muscle, sternoclavicular tenodesis using the sternal head of the sternocleidomastoid muscle [27] and sternoclavicular joint reconstruction with the semitendinosus, hamstrings, palmaris longus, or allograft tendon [28–30]. In these surgeries, either the amphiarthrodial function of the sternoclavicular joint is sacrificed to achieve adequate fixation, or postoperative complications, such as internal fixation displacement, reduction loss, and infections, are usually observed because of complex surgical manipulation. Currently, there is no well-recognized internal fixation equipment suitable for posterior sternoclavicular dislocation and medial clavicle fractures.

Behind the proximal clavicle are important structures such as the apex pulmonis, mediastinum, and the subclavian artery and vein; thus, surgeries in this area are high-risk. In addition, SCD treatment does not use a special steel plate, and it usually uses internal fixation instruments designed for other diseases [31]. Generally, the risk for surgical complications is high; therefore, safe and effective internal fixation devices and surgical procedures for SCD are required. Alternatively, an external force is directly applied to the front of the proximal clavicle, which is pushed behind the sternum, resulting in posterior dislocation of the proximal clavicle within the sternoclavicular joint. Because closed reduction of posterior SCD mostly requires general anesthesia and that patients’ CT scans indicated that neurovascular structures are in close proximity to the dislocated clavicle, we did not attempt closed reduction in patients with posterior SCD, instead opting for open reduction.

### Advantages and disadvantages of the sternoclavicular hook plate in the treatment of posterior SCD

The sternoclavicular hook plate is a special type of steel plate designed to treat SCD; the treatment of posterior SCD has obtained a good curative effect, as follows: (1) The sternoclavicular hook plate’s mechanical stability is adequate due to its sternal bone hole activity while preserving the sternoclavicular joint micro-motion; thus, patients can perform early functional exercises. Iin this study, both the Constant–Murley and Rockwood sternoclavicular joint scores suggested that postoperative acromioclavicular and sternoclavicular joint functions could be effectively restored. (2) When a sternoclavicular hook plate was used, the surgical incision exposed only a third of the medial clavicle and a part of the sternum, and the insertion point of the sternocleidomastoid muscle was well preserved. Furthermore, it had less interference on soft tissues and blood vessels around the joint; therefore, the sternoclavicular hook plate was beneficial for dislocation healing after surgery. (3) The hook part of the sternoclavicular hook plate is close to the rear of the sternoclavicular joint in an arc, effectively avoiding injury to important blood vessels and organs behind the proximal clavicle. The threaded head of the hook can be effectively fixed on the anterior edge of the sternum by adding nuts and spacers to avoid complications such as posterior loosening and displacement of the internal fixation.

Attention should be paid when using internal fixation in elderly patients with osteoporosis. When the sternal end is drilled and inserted into the hook part of the plate, the activity of the upper limb is transmitted to the hook plate through the clavicle. The hook head becomes a major force release point that forms an axis of activity, and bone absorption occurs around the hook head on the sternal side. If vigorous activity is performed early, the loose sternum may not be strong enough to block the trending force of clavicle dislocation, which can lead to a cut and result in failure of internal fixation.

### Treatment experience

Posterior SCD often occurs in highly violent injuries during athletic events or motor vehicle accidents [13, 32]. Most commonly, an indirect force on the posterolateral shoulder forces the lateral clavicle anteriorly and levers the medial clavicle posteriorly. Less commonly, a significant posterior force is applied to the medial clavicle [33, 34]. Early diagnosis of this disease is often obscured by the presence of other sites such as the head, chest, and abdomen. After the patient’s condition is stabilized, further comprehensive examination is necessary. Traditional internal fixation methods are prone to losses, which can lead to fixation failure. The end of the sternoclavicular hook plate was inserted from the rear of the sternum through a sternal hole and was hooked to the sternum. After completion of internal fixation, the entire structure maintains the original micro-dynamic environment of the sternoclavicular joint and meets the biomechanical requirements. Treatment experiences are as follows: (1) When the hook part of the sternal hook plate is used to enter the sternum, the lymph and veins behind the sternum should be gently separated with a long and bent nerve dissector. (2) When drilling the sternum and clavicle, our specially made drill jig should be used to avoid excessive drilling and damage to the nerves, blood vessels, and organs behind the bone. (3) In the treatment of posterior dislocation, a thin spacer and locking nut must be installed at the head of the thoracic hook plate to prevent backward displacement.

### Limitations

The clinical incidence of posterior SCD is low. This study did not compare the sternoclavicular hook plate internal fixation method with other internal fixation methods. At the same time, prevention of posterior SCD with sternoclavicular hook plates in patients with osteoporosis requires further investigation. With further research, better results could be achieved in patients with posterior SCD.

## Conclusions

In conclusion, the novel sternoclavicular hook plate achieved satisfactory results in the treatment of posterior SCD. Therefore, it is a convenient and safe internal fixation method. Moreover, the micro-motion function and stability of the sternoclavicular joint were preserved, allowing patients to perform early functional exercises postoperatively.

## Data Availability

The datasets used and/or analysed during the current study are available from the corresponding author on reasonable request.

## Abbreviations

SCD: Sternoclavicular dislocation
CT: Computed tomography
ROM: Range of motion
